# Strategies in overcoming racial and socio-cultural differences in the learning environment of post-graduate medical specialty training in South Africa

**DOI:** 10.15694/mep.2018.0000062.1

**Published:** 2018-03-13

**Authors:** Aye Aye Khine, Nadia Hartman

**Affiliations:** 1 Sefako Makgatho Health Sciences University; 2 University of Cape Town

**Keywords:** Racial, Socio-cultural, Medical Speciality, Resilience

## Abstract

This article was migrated. The article was marked as recommended.

Study problem

The training of postgraduate medical students in the multi-racial landscape of South Africa has faced challenges given the need for relationships in personal mentoring and learning through legitimate participation in the community of practice (CoP), as part of cognitive apprenticeship training. A high failure rate in the exit examination had stimulated interest into understanding the nature of the learning environment.

Aim and objectives

The study explored conceptions of former students in a medical specialty program regarding the nature of racial and socio-cultural diversities in their learning environment, influences on learning, and how they responded to them.

Methodology

A qualitative enquiry using in-depth interviews with semi-structured open-ended questions and thematic analysis with a social constructionist approach of epistemology used for data analysis and interpretation.

Findings

Students conceived race, language, departmental culture and social identity as barriers in their learning. The lack of structured formative training with feedback, evaluation, personal mentoring, and supervision also emerged. Through resilience, adaptability, and maturity qualified students overcame these difficulties.

Conclusion

Current and future students may benefit by developing resilience when dealing with racial and socio-cultural differences, and findings support the inclusion of cultural competence and a multi-lens approach in medical specialty curricula.

## Study problem and context

This study was based in a medical specialty programme with students who had lived and studied through the latter years of the apartheid period in South Africa, and thus were exposed to racial and socio-cultural divisions and inequalities, and through the twenty years after apartheid as the diverse races came together to share the learning platforms. This medical specialty programme is offered at the seven medical universities across South Africa. The training period is four to six years and the students have spent a minimum of two years in the clinical practice before they joined the program. They are employed by the service provider as trainee-workers whilst enrolled with academic departments. The specialty curriculum was blueprinted in 2013, but the model of training was not clarified. Globally, training of a medical specialty has been postulated as cognitive apprenticeship in developing novices into experts, and such a model requires mediation by a personal mentor, with participation in the CoP learning through situated contexts, both of which place the importance of meaningful engagement and a close relationship between the trainer and the student ( Collins, 1987; Cruess, Cruess & Steinert, 2008; Bezuidenhout, Cilliers, Heusden, Wasserman, & Burch, 2011; Genzen & Krasowski, 2007).

The selection and admissions policy changed during post-apartheid transformation and put a focus on training Black African doctors, but the consultant trainers were still by majority Whites and Indians of the apartheid education era, thus was created an interface of racial and socio-cultural diversities. The majority of specialty students were Black Africans with a few Indians depending on the geographical area with its particular population profiles. This led to the situation where previously disadvantaged students learnt from consultants from the previously dominant racial group ( Lumadi, 2008; Jansen, 2004; Sehoole, 2012).

In the cognitive apprenticeship training model, consultants are required to provide content for expertise development, to effectively mediate the students (also known as registrars) to facilitate their cognitive development through using situated learning opportunities and encourage the registrars to legitimately participate in the informal discussions and problem solving of departmental community of practices. Effective learning would require active engagement, in-depth discussion between the peers and the consultants, and discussions outside of the formal training programme that form an important part of learning in medical specialities. Relational aspects with empathy, trust and mutual respect between the student registrars and the trainer consultants become crucial. A high failure rate in the recent years after implementation of the transformation policy in the medical specialty alarmed the stake-holders to probe into underlying reasons to rescue the situation. Given the landscape of historical racial and social divisions in South Africa’s higher education system, a study was proposed to explore the students’ conceptions of diversities in their learning environment.

The framework is based on the cognitive apprenticeship model of training ( Collins, 1987) for intermediary novices (named as such because they are already medical doctors studying to be specialists) into expert specialists. The model is implemented through socio-cognitive and socio-cultural learning platforms, the former being mediation by the expert mentor (Vygotsky, 1987) and the latter being learning through situated contexts within the CoP ( Lave & Wenger, 1991). It is expected that the mediation it will be done through: Modelling as the expert shows the registrars how to perform a task; Coaching where the consultant observes and facilitates while the registrar performs a task; Scaffolding as the consultant provides a step-wise approach under supervision (tailoring tasks according to the individual’s need) and guidance, then slowly fade the guidance away; Articulation as the consultant explains how he/she thinks about solving the problems and encourages registrars to verbalise their understanding /conceptualising and thinking (which is also referred to as thinking aloud); Reflection where the consultant enables registrars to compare their performance with others, self-evaluate performance and also judge against the expected outcomes; and Exploration where the consultant asks registrars to create simulated problems and solve them. These processes reflect the mediation of the more knowledgeable other in the zone of proximal development, and the primary importance is the relationship between the mentor and the mentee that determines the degree of interaction, engagement and participation.

Relationships are again influenced by the attitudes, behaviours and perceptions of people towards each other. These are thought to be shaped by what people went through in their previous environments and how these shaped them. This was the concept of Bronfenbrenner (1977) in explaining the Human Ecology theory’s contribution to an individual’s belief, perception, attitude and behaviour. The intricacy of relational aspects in the training of medical specialities becomes more complex within the context of South Africa’s history of apartheid that both the participants, peers and the consultants lived through or were subjected to. Thus, a study was conducted to unpack and understand the complex phenomenon whereby in the recent past some Black African students had failed the exit examinations and left the formal training program, whilst some of them managed to qualify.

### Study Aim

Based on the theoretical framework, the study aimed to explore the conceptions of two student groups (those who had left the specialty programme, and those who had recently qualified) regarding the nature of racial and socio-cultural diversities in their learning environment, the influences on their learning, and how the students responded to them.

### Methodology

Under the constructivist paradigm and relativist ontology, the study employed a qualitative enquiry using individual in-depth interviews with semi-structured open-ended questions to unpack the participant’s experiences, perceptions, constructions and conceptions around the racial and socio-cultural diversities in their previous learning environments including the medical specialty training.

The interviews were audio-recorded, transcribed and coding was done by deductive and inductive approaches of thematic analysis. The data interpretation was done in the social constructionist approach that analyses the conceptions co-constructed by the students as peers during their training, and also by the Researcher, participants and the co-analyst.

### Study population

The sample consisted of students previously enrolled in the medical specialty of interest in this study across six universities’ academic departments in South Africa. There are seven universities in South Africa, though the Researcher’s department was excluded for ethical necessities. The students of focus in this population were the ones who had failed the exit examination, and those who had been successful and qualified in the period 2012 to 2016. The total number was twelve with six in each group. This period was taken as such in order to keep the data recent and relevant to the current context. From the first group five and from the second group four agreed to participate in the study.

### Ethical considerations

Anonymity was maintained by keeping the participants’ identity, as well as the training location, completely anonymous by using pseudo-names and not mentioning the gender, age and location of the participants in any record or submissions. All participants signed a confidentiality commitment, and all records were treated with the utmost care for confidentiality. The Researcher has restrained herself from participating in the exit examination either as a convenor or an external examiner for the two years of the study period. Participants were given freedom to leave the study without any consequences in their learning. To avoid any conflict of interest previous students who studied in the Researcher’s department were excluded.

### Dealing with biases and validity

Drawing on the recommendations of Lincoln & Guba (1989), the Researcher’s biases were reduced by self-awareness and reflexivity throughout the process of interviews, data coding, analysis and interpretations. The audio records were transcribed by a professional transcriber and data analysis and interpretations were done by the Researcher, and also one independent co-analyst in parallel.

As built-in internal checks during interviews, the practice of repeating, clarifying and asking further probing questions reduced biases and misrepresentations. During data analysis member checking was done via email for coded transcripts. To improve data validity data triangulation was performed by recruiting former students of various universities across the country to compare and contrast across the academic departments and by various participants. Race and gender of participants were not inclusion criteria so that bias was reduced. A social constructionism approach of data interpretation, whereby the emergence of sub-themes and themes are co-constructed, also promoted a multi-lens approach that further reduced the biases.

**Figure 1.**
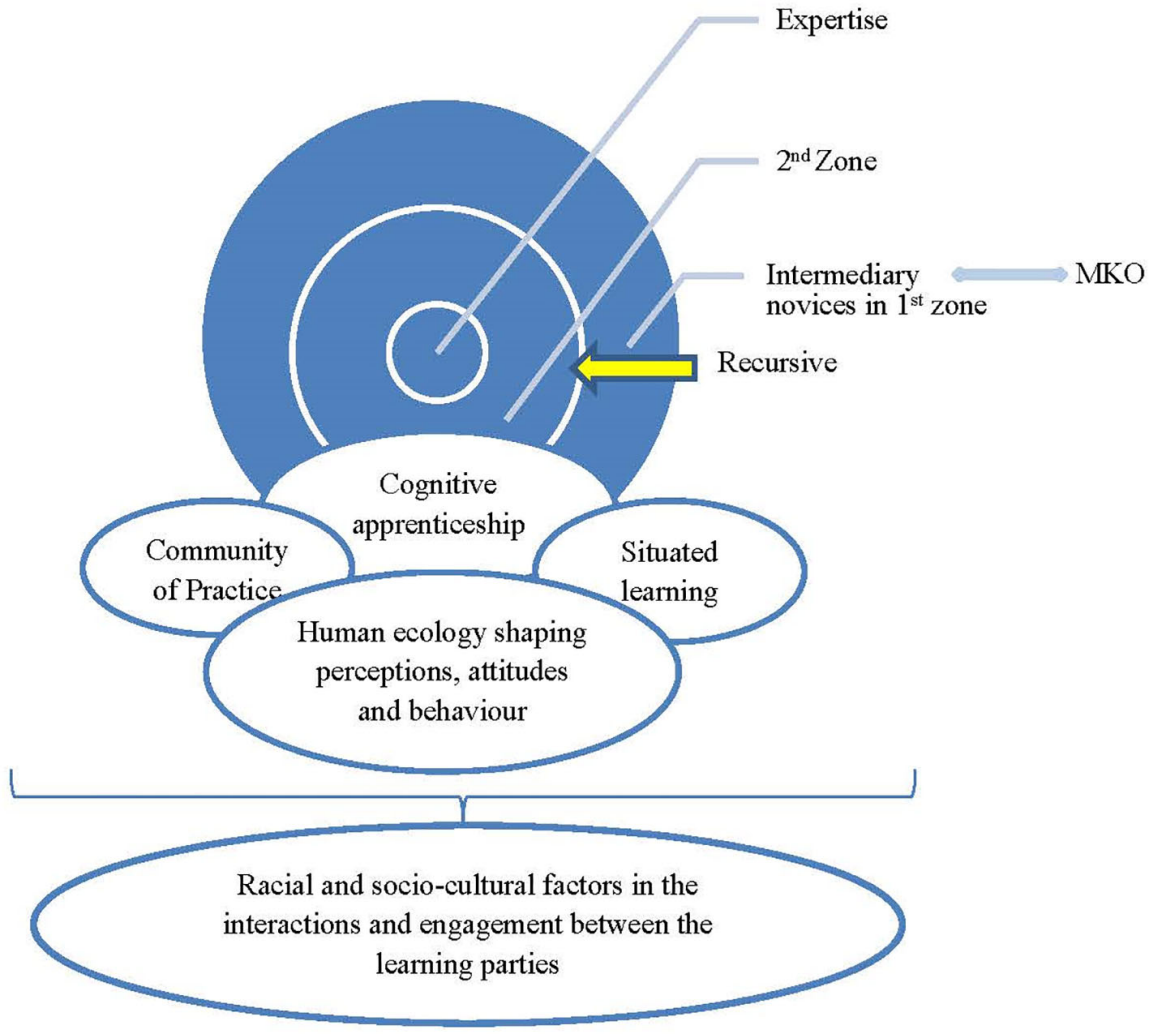
Diagram depicting relationships of theories and their concepts in this study

## Findings

Four major themes emerged from the data.

### Theme 1 - Constructing ‘racial’ and ‘social-cultural’ diversities as differences that posed barriers in learning

#### Sub-theme: Constructing ‘race’ as a barrier in learning

The construction of race had a strong influence from the apartheid times, as the participants in this study and their families had lived through the system. Black registrars mentioned being underrated by assumptions of being less academically prepared and felt that they had to make an impression early in the programme to establish themselves as being ‘smart’, so as to have a positive learning experience. One participant (Black/qualified) stated that *“I think generally, the nature of the registrar programme is that if you are a Black person, it’s a times two. Meaning you need to work two times harder than other races, that’s what I’ve realised.”* Another participant (Black/left) believed that one should maintain self-identity, beliefs and ways of living and not be transformed and related *“I believe never to succumb to being demeaned by someone, you know for who you are.. for the colour, for the way you talk..that’s your own identity”.* Whilst some participants felt that this was a barrier to their learning, others were able to overcome the differential treatment and qualify.

#### Sub-theme: Constructing ‘language’ as a barrier in learning

Black South Africans participants found the language barriers of Afrikaans and English difficult whilst attending universities for undergraduate medicine, and conceived this as a barrier in their learning. As one of them mentioned *“It was a huge shocker, not necessarily because it was a different language, but the fact was the expectation was that I had to know Afrikaans for me to learn medicine.”* Despite the challenges, all of the participants overcame the language barrier in their undergraduate training by various resources such as peer support and lecturer interactions. During postgraduate training there was another dimension in the language barrier that was the expected academic language proficiency. A participant (Black/qualified) was not able to express fluently in English and explained *“I talked slowly as I had to translate English to vernacular and back there again”.* Another Black participant reported the struggle of understanding the English accent of an Indian consultant, and complaints about her African accent in speaking English during academic presentations. Most Black South African students conceived that they were prejudiced through the choice of the Afrikaans language, or by their proficiency of the English language.

#### Sub-theme: Constructing ‘culture’ as a barrier in learning

All participants regardless of race experienced the culture of their academic departments as demotivating with a lack of support, based on the harsh comments from the consultants during formal presentations, and the prevailing expectations of self-study without supervision and guidance. Those who left the program felt that they were lost, peers were divided by power struggles, and had experienced a lack of interest in mentoring amongst the consultants in the department. These participants experienced being subjected by their consultant coordinator, and felt that their own identity was threatened. Two qualified participants had not associated these aspects with racial domination and conceived the departmental culture as emanating from individual’s personalities.

#### Sub-theme: Constructing feeling ‘excluded’ by social status

During postgraduate training, all participants had previously been medical practitioners and performed community service, whilst others had already worked as medical practitioners for considerable years and accumulated more wealth. Family status also varied amongst participants as some were married with children, and others remained single. Some felt these differences posed challenges in how they related to each other, and to the consultants. They believed that they were treated differently due to their social identities, and that hindered a positive learning environment.

### Theme 2: Relationships in the learning environment shaping learning

Emerging from the interview transcripts from all participants was the ability to form strong relationships in the schooling years with peers from the same background and diverse teachers, which supported their learning and growth. They all had a loving, caring and good relationship with their family and teachers that supported their learning throughout the school years contributing to their self-confidence and success to enter the various medical schools.

The majority of the participants had to move away from home to attend medical schools as they lived far from the seven medical schools in the country, and were exposed to socio-cultural diversities. Participants managed to form relationships with peers to learn in group discussions using each other’s support, although they were mostly with peers of the same background. A participant commented that *“we used maybe expertise or guidance of guys who were one class ahead of us”* as they understood the difficulties facing them. Another participant (Indian/qualified) attending a medical university with diverse races mentioned that *“there was a lot of interaction with all the races, and we sort of learned from each other”.* The participants’ constructions reflect that during undergraduate medical training they overcame the difficulties related to language, and access to resources and support.

Whilst during postgraduate training there were disjointed expectations between the registrars expecting one-on-one mediation, and the consultants promoting self-learning. Behavioural expectations from the consultants towards registrars such as submissiveness and obedience caused tensions, with some registrars having maintained being assertive, if that was their personality. Participants believed that these demands were underpinned by power and control issues from the consultants, and this is what strained their relationships. Open and honest discussions could not take place due to the lack of forming relationships, as a participant mentioned *“because we had no relationship, and that was identified as a shortcoming in our training”.*

The qualified participants reported having to resort to self-directed learning and formed study buddies with peers from the same department or from other universities, who were also preparing for the exit exam. They found this cooperation especially helpful to diversify their learning approaches, keep awareness of trends, and different perspectives. Students who qualified refused to give up, and found alternative ways to form relationships with consultants. Through demonstrating self-initiation in learning they managed to convince the consultants to provide guidance, or in one participant’s case a full mentorship, that helped them in eventually passing the exit examination.

### Theme 3: Challenges in the learning process

Participants’ conceptions revealed multitudes of factors that are critical for cognitive development, and yet not provided in their formative training such as: curriculum awareness and guidance, alignment of day-to-day practice to the assessment criteria, and in the structure of formative training. Elements of a curriculum such as clarity in programme outcomes, learning and training methodologies, supervision and mentoring, formative assessments and feedback, learner’s motivation, and roles of the mentor and roles of mentees were not part of the formal curriculum published by the authorities for this discipline. The blueprinted summative assessment was not supported by the formative training, as there was a lack of structure in the latter. One participant recalled that *“specifics of expectations and how to go about them and what you needed to cover, only came up later”*, and felt this knowledge came rather too late. Departments tried to set up internal mock exams near the end of the training, but participants felt they were not prepared for the level of assessment in these tests and there was no time left to bounce back from the failures in the mock tests.

In this specialist discipline, formative learning can only be achieved if there are constructive interactions during the academic seminars, journal discussions and case discussions. These are formal learning platforms, and the informal learning is through ad-hoc discussions on the problems and cases presented to the department functioning as a CoP. These interactions promote deeper learning and facilitate the development of skills in analytical problem solving reaching meta-cognitive learning for the students. Entry to the CoP by the registrars needs to be facilitated by the senior members, and their peripheral legitimate participation in the discussions should be promoted and encouraged. Practical skills can also be achieved during routine day-to-day patient services, but they needed to be well supervised and monitored/evaluated. These are an integral part of the curriculum and should have constituted the most important component that impacts learning and preparation for the summative assessment.

All participants had experienced a lack of interactions in their formal presentations and did not receive supervision in their day-to-day service provision. Most departmental CoPs did not exist, or if they did junior members felt discomfort and were either unable to participate. However, the qualified participants found alternative ways to approach consultants with problems identified by themselves during service provision, and to request the consultant’s opinion in solving these problems, thereby changing the self-learning to partially guided learning. One qualified participant was offered a mentorship by the external consultant who had noticed the registrar’s potential whilst being unsuccessful in the previous exit examination.

### Theme 4: Overcoming challenges

With the group that qualified different approaches to adaptation and finding alternative solutions were evident when faced with such adversities as a lack of curriculum, an absence of mentorship and training, power and relationship issues with peers and consultants. One participant (qualified) felt that *“Maybe it helped to train when you’re slightly older, because you already knew that’s what you wanted, and therefore you had to persist.”* and believed that maturity relates to open-mindedness and being able to focus on the goal overlooking other distractions. Another participant (qualified) reflected on the approach to dealing with racial and socio-cultural difference was to have a ‘thick skin’ and focus on what she had come there for and she stated that *“I learnt to be tough and resilient”.*

Recursively reading all the transcripts, it was clear that although the challenges were similar for all participants, the ways the participants see the challenges and how they responded were quite different. Some constructed the challenges as being rooted in their personal differences with the consultants, such as race or personality, and some as being part of different cultural ways of doing things. Participants that left expressed feelings of being put down and humiliated by the harsh remarks from the consultants, and could not transcend these challenges. They rather felt that their self-esteem and confidence was attacked. The qualified participants also felt the same, but were able to discern these as distractions, and took the negative remarks as a source of motivation to change something in their learning approach. They constellated resiliency from multiple angles such as: maturity open-mindedness, an ability to focus, to discern what to engage with and what to overlook, to self-direct deeper learning, to initiate enquiry and remain engaged, to maintain a positive outlook on different cultures and ways of interactions, to form relationships through mutual respect and tolerance despite differences, and to take responsibility in one’s own learning.

The participants that left the training programme had also demonstrated resilience in their own way as being able to endure the entire training period, and some continued to inspire by sitting for the exit exam even after leaving the programme. They reflected that they regretted not striving for unity amongst their peers, retrospectively having realised the importance of this. Some believed that the outcome may have been different had they tried to conform to the requirements of the department cultures.

### Theme 5: Being shaped by the environments

The data showed that participants responded to the similar challenges in variable ways, and drawing on the concept of Bronfenbrenner (1977), perceptions, attitudes and behaviours are shaped by previous environments. Family upbringing as a micro-environment; schooling, community or society upbringing as a meso-environment; and country history and socio-political dynamics as a macro-environment; can shape the individual’s preconceived ideas and beliefs. It was noted that the perceptions of their preconceived ideas were more pronounced and affirmed in the participants’ attitude during their postgraduate training. A participant (Black/qualified) who had grown up in a family who had lived and worked on a farm during apartheid times, provided a basis for her lack of trust in this different other race as she mentioned *“we were always protected by our parents from going near to Africana people or speaking their language”.* Another participant (Black/left) was told by his parents *“try and do as much as you can, but don’t lose your own beliefs and identity”.* Similarly, a participant’s (Black/left) whose parents had been forced to leave South Africa and go into exile in a neighbouring country, on returning experienced difficulties and explained that *“I was taught to always think and talk for myself so I cannot grow in a place where I cannot voice what I believe”.* One participant (Indian/qualified) believed that her attitude towards respect for others came from her family upbringing and mentioned “ *I think it’s a lot to do with upbringing, in terms of being compassionate to another person, to be kind to another person and also it’s very much to do with personality*”.

It is apparent that whilst some participants were supported by their family upbringing micro-environment to adapt to the environment and its requirements posed by the different racial groups, others had carried the scars of their upbringing with them and may have experienced this as a barrier that they were unable to overcome.

## Concluding thoughts

The findings of this study may directly benefit current students who have to immerse in these diversities, especially if they perceive them as differences affecting their learning. However, resilience and the associated attributes reflect a personal capacity shaped by the individual’s past experiences and environments, and may not be possible to transfer to others. Future research should look into how students can learn to develop resilience, especially when dealing with consultants and peers from a different and previously dominant background. It may be possible through mentoring by those who have already successfully demonstrated resilience in learning, or through peer support groups. The conclusions however cannot be generalised for other medical specialities as there may well be different characteristics of students and consultants in other cohorts. Thus, there is an opportunity to explore further how students in other medical speciality cohorts perceive, construct and conceive the different dimensions of racial and socio-cultural diversities in their environments, how they impact on their learning, and approaches they have used so far to deal with them.

The main limitation of the study was that the consultants were not included in order to protect and provide anonymity to the students, and this prevented the Researcher having an opportunity to learn the perspectives from the consultants.

Nonetheless, the Researcher hopes that the findings may pave the way for introspection in both the students and the consultants of this programme, and other similar medical specialty programmes, allowing them to negotiate their way (even silently) to develop more meaningful relationships, as they understand the essential benefits of such in the learning process.

## Take Home Messages

• Race is a social and cultural phenomenon, and it is continuously being reconstructed based on how the environment shapes one’s perception, changing circumstances, culture and practices of institutions ( Dolby, 2001).
• Drawing on the need to use the cognitive apprenticeship model in the medical specialty training, the relationships are in the pivotal role of facilitating learning and development.
• Consultants to note the importance of understanding the student’s background, culture, and nature (personality) in making attempts to reach out to them, and to shape their learning and development.
• Ozbay et al., (2007) described six essential behavioural factors that can help sustain one’s strength and integrity during stress: a positive attitude explained as optimism and a constructive outlook to criticism, a sense of humour, flexibility in learning, ability to discern right from wrong, inclination for physical activity, and availability of social support and relationships with role models.
• Resilience can be trained and developed in amendable people, who are going through difficulties in their life. This can be done by workshops, support groups or personal mentoring ( Proudfoot, Corr, Guest and Dunn, 2009).

## Notes On Contributors

Dr Aye Aye Khine is a consultant specialist, trainer and examiner of multiracial students for a medical specialty (in this study). As a naturalised citizen of South Africa, she did not grow up under the apartheid regime, and is therefore racially and socio-culturally neutral in this environment.

Dr Nadia Hartman convenes the Masters and Doctoral Programmes in the Department of Health Sciences Education at the University of Cape Town. Her speciality is curriculum development in health sciences/professions education, with a special focus on socially responsive education and training.
