# Supplementary material for: Strategies in overcoming racial and socio-cultural differences in the learning environment of post-graduate medical specialty training in South Africa
Source: MedEdPublish (2016). 2018 Mar 13;7:62. [Version 1] doi: 10.15694/mep.2018.0000062.1 (PMC10711947; doi:10.15694/mep.2018.0000062.1)
Supplement: Peer review history

# Reviewer report: Reviewer response for version 1

**DOI:** 10.21956/mep.19533.r28919

**Published:** 2020-01-05

**Authors:** Peter Thomas Cartledge

**Affiliations:** 1 ale University, Rwanda Human Resources for Health (HRH) Program, Kigali, Rwanda

This review has been migrated. The reviewer awarded 4 stars out of 5 An important piece. I would have enjoyed seeing even more insight into how some of these challenges could be overcome.

Reviewer Expertise:

NA

No decision status is available

# Reviewer report: Reviewer response for version 1

**DOI:** 10.21956/mep.19533.r28921

**Published:** 2019-05-08

**Authors:** Ken Masters

**Affiliations:** 1 Sultan Qaboos University

This review has been migrated. The reviewer awarded 3 stars out of 5 An interesting paper dealing with the high failure rate in a postgraduate medical program in which most of the students came from previously disadvantaged racial backgrounds, and in which most of the teachers had more advantaged racial backgrounds. The paper begins the process of exploring the impact of racial and cultural differences on the students’ performance.A particular strength of the paper is the fact that both successful and unsuccessful candidates were interviewed. The authors are further commended on relating their work to the theoretical background. Two issues that lower the value of the paper:• I understand that the issues discussed are delicate and sensitive, but these concerns hamper the research, and appear to prevent deep exploration. The few as possible identifying aspects of the interviewees leaves the reader with a feeling of reluctance on the part of the researchers to probe more deeply. There is no doubt that racial classification is an over-riding characteristic in the inequalities, but within those, there are others also, such as gender, age and socio-economic background, which can cause fundamental differences in how different people with the same racial classification are affected by external events. By not addressing these and other aspects, or touching on them very briefly only (not even to mention the gender and age of participants), the researchers have left the readers with a very superficial impression. An opening presented itself in the discussions of language, and it would have been beneficial to the research if the researchers had probed the links to cultural prejudices and power dynamics associated with the different languages, particularly given the dominant language of the apartheid government, and the deep political associations and implications of the school language of instruction. The researchers are perhaps confined by the permissions of the environment, but, until qualitative research of this nature is able to delve far more deeply into these issues, there will always be a lack of understanding of all the issues, and taking steps to solve the problems will be severely hampered from the outset. • The small sample size: while small sample is not always a problem in qualitative research, it would have been mitigated somewhat if the authors had chosen some form of saturation process to determine the sample size. Failing that, it would have been useful if the researchers had given some idea of the extent to which theme saturation had been reached.So, a useful start, but one would like to see a much deeper exploration of the issues at hand

Reviewer Expertise:

NA

No decision status is available

# Reviewer report: Reviewer response for version 1

**DOI:** 10.21956/mep.19533.r28920

**Published:** 2018-03-23

**Authors:** Trevor Gibbs

**Affiliations:** 1 AMEE

This review has been migrated. The reviewer awarded 5 stars out of 5 A very well written paper that attempts to explore very real problems and challenges. I thought that the paper fully exposed the difficulties of finding an ideal learning environment and how we need to take the diverse situations that learners are facing into consideration when assessing how fruitful that learning has been. A paper all involved in curriculum planning need to read

Reviewer Expertise:

NA

No decision status is available
